# Surgical treatment of a giant unruptured aneurysm of the noncoronary sinus of Valsalva: a case report

**DOI:** 10.1186/s13256-016-1040-2

**Published:** 2016-09-19

**Authors:** Edvin Prifti, Fadil Ademaj, Arben Baboci, Edmond Nuellari, Aurel Demiraj, Dariel Thereska

**Affiliations:** 1 Division of Cardiac Surgery, University Hospital Center of Tirana, Tirana, Albania; 2 Division of Cardiology, Gjakovo Hospital, Rr. Prizren, Gjakovo, Kosovo

**Keywords:** Unruptured aneurysm, Coronary sinus of Valsalva, Patch repair

## Abstract

**Background:**

A sinus of Valsalva aneurysm is a rare cardiac anomaly which may be acquired or congenital. The main associated symptoms are conduction disturbances, myocardial ischemia, and syncopes.

**Case presentation:**

In this report we describe a 52-year-old Albanian woman from Kosovo with an unruptured aneurysm of 74×60 mm of the noncoronary sinus of Valsalva presenting dyspnea, jugular distension, and tachycardia due to cardiac compression. She underwent successful closure of the orifice and sinus remodeling with a Dacron patch.

**Conclusion:**

To the best of our knowledge this is the largest reported isolated unruptured aneurysm of the coronary sinus causing severe compression of the cardiac chambers undergoing successful surgical correction.

## Background

A sinus of Valsalva aneurysm is a rare cardiac anomaly which may be acquired or congenital, intracardiac or extracardiac [1]. In 65 to 85 % of cases a sinus of Valsalva aneurysm presents in the right coronary sinus, in 10 to 30 % of cases it presents in the noncoronary sinus, and in <5 % of cases it presents in the left coronary sinus [2]. A sinus of Valsalva aneurysm rarely presents with symptoms; if there are symptoms, they can be symptoms of conduction disturbances, myocardial ischemia, syncopes, and symptomatic cardiac dysfunction [3]. We describe a patient with an unruptured giant (the largest ever reported in the literature) aneurysm of the noncoronary sinus of Valsalva presenting symptoms of cardiac compression who underwent successful surgical repair.

## Case presentation

**Fig. 1:**
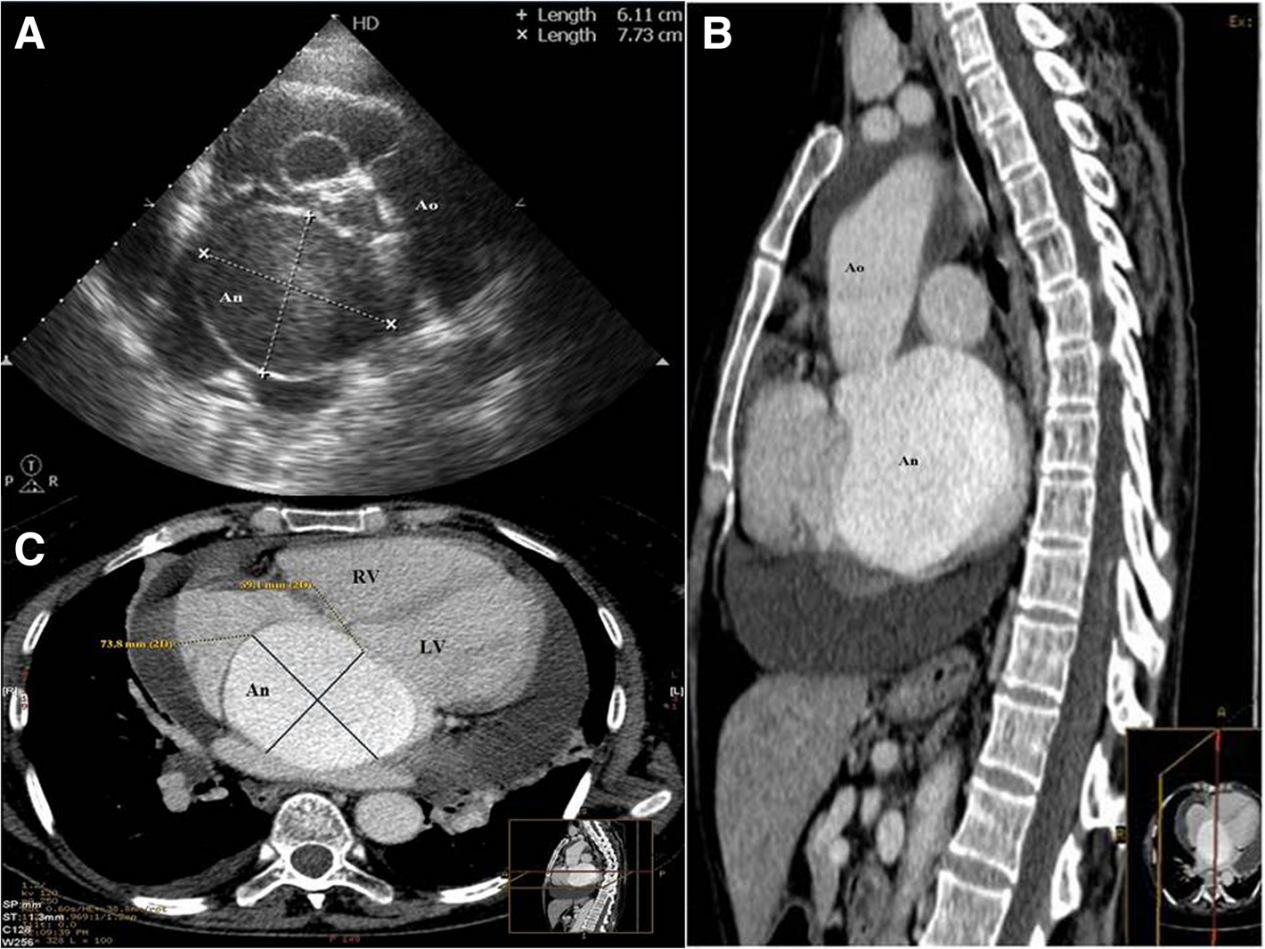
**a**. Transesophageal echocardiography demonstrating a giant aneurysm of the noncoronary sinus. **b**. Unruptured aneurysm of the noncoronary sinus. **c**. A giant aneurysm of the noncoronary sinus measuring 74×60 mm. *An* aneurysm of the coronary sinus, *Ao* aorta, *LV* left ventricle, *RV* right ventricle

A 52-year-old Albanian woman from Kosovo presented to our hospital with dyspnea, tachycardia, and jugular distension. On admission she was in New York Heart Association class III, with normal blood pressure and absence of aortic murmur. She was in sinus rhythm and a chest X-ray demonstrated cardiomegaly with right atrium enlargement. A transthoracic echocardiography demonstrated increased pericardial effusion of almost 35 mm and a giant mass above her left atrium communicating with the ascending aorta. A transesophageal echocardiogram showed a large unruptured aneurysm/pseudoaneurysm 61×77 mm of the noncoronary sinus of Valsalva, without thrombosis or shunts, above her left atrium (Fig. 1a). Her aortic valve was competent, without annular enlargement. A cardiac contrast-enhanced angio-computed tomography (CT) scan confirmed the presence of a giant unruptured aneurysm of the noncoronary sinus of Valsalva of 74×60 mm diameter free from thrombotic lining, which was compressing her right atrium (Fig. 1b, c). The presence of a significant amount of pericardial effusion indicated a possible rupture of the aneurysm of the sinus of Valsalva or the presence of a pseudoaneurysm; therefore, we recommended urgent surgical repair.

**Fig. 2:**
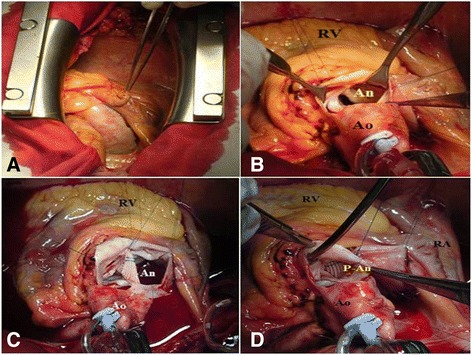
**a**. Intraoperative view of the unruptured aneurysm located superiorly to the left atrium. **b**. The visualization of the orifice of the unruptured aneurysm of the noncoronary sinus. **c**. The implantation of the synthetic patch on the orifice. **d**. Total closure of the orifice of the aneurysm. *An* aneurysm of the coronary sinus, *Ao* aorta, *p-AN* prosthesis of the aneurysm; *RA* right atrium, *RV* right ventricle

**Fig. 3:**
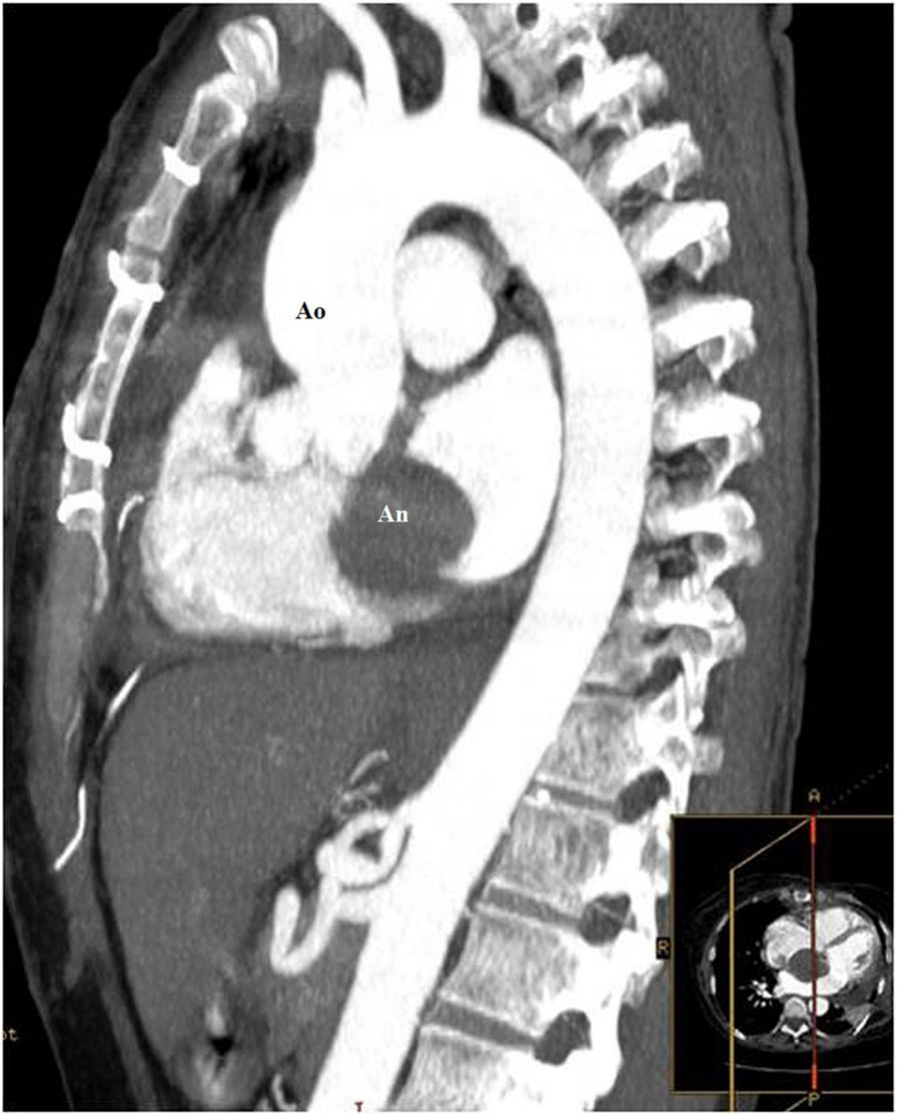
Postoperative contrast-enhanced angio-computed tomography demonstrating total obliteration of the aneurysmatic sac. *An* aneurysm of the coronary sinus, *Ao* aorta

She underwent femoral artery and vein cannulation and on cardiopulmonary bypass a sternotomy was performed. Her pericardium was then opened. An unruptured aneurysm of the noncoronary sinus of Valsalva was identified (Fig. 2a). On intraoperative examination, a thin-walled unruptured aneurysm of the noncoronary sinus of Valsalva expanding above the roof of her left atrium, compressing and partially gaining adhesions with her right atrium was identified. Her aorta was clamped and her heart was arrested with anterograde cardioplegic infusion. An anterior oblique aortotomy was performed. The orifice of the unruptured aneurysm of the noncoronary sinus of Valsalva was identified (Fig. 2b). The aneurysm was opened and carefully inspected for any possible communication. A Dacron patch was prepared to remodel the noncoronary sinus, which then was sutured with a continuous Prolene 4/0 suture (Fig. 2c, d). Then the aneurysmatic sac was closed from outside above the orifice of the aneurysm. A pathological examination of the resected unruptured aneurysm of the noncoronary sinus of Valsalva revealed conspicuous mucoid deposits, loss of elastic fibers and eosinophilic infiltration. Her postoperative course was uneventful. At 1 month after surgery, a contrast-enhanced angio-CT demonstrated a totally thrombosed cavity of the previous aneurysm which had non-communication with her aorta or any other cardiac chamber (Fig. 3). At 1 year after surgery, she was doing well and an echocardiographic examination revealed mild aortic insufficiency.

## Discussion

To the best of our knowledge there are no previous published data of an acquired unruptured aneurysm of the noncoronary sinus of Valsalva of such dimensions, which first presented as a cardiac tamponade with the additional symptoms and signs that are typical in these settings, such as dyspnea, tachyarrhythmias, and jugular distension. The most frequently found symptoms of an unruptured aneurysm of the sinus of Valsalva are exertional dyspnea [3], palpitation, chest pain associated with coronary anomalies [4], acute coronary syndrome [5], syncope [6], cardiac murmurs [7], or totally asymptomatic [8].

Sinus of Valsalva aneurysms can be acquired secondary to infections, degenerative conditions, or trauma. Most cases are congenital due to a defect in the aortic wall, more precisely of the continuity between the aortic media and aortic annulus. The essential lesion of a sinus of Valsalva aneurysm is separation of the aortic media of the sinus from the media adjacent to the hinge line of the aortic valve cusp. The differential diagnosis of sinus of Valsalva aneurysm includes isolated pseudoaneurysm of the sinus of Valsalva. In the case of a pseudoaneurysm the orifice is apart from the aortic annulus, which is not the case in a sinus of Valsalva aneurysm where the orifice of the aneurysm is adjacent to the aortic annulus. In a pseudoaneurysm of the sinus of Valsalva there are no specific histologic changes in the aneurysmal wall. An association with Marfan and Ehlers–Danlos syndromes, bacterial endocarditis, syphilis, and mycotic infection has been reported [9]. In our current case, the medial wall of the unruptured aneurysm of the noncoronary sinus of Valsalva showed conspicuous mucoid deposits, loss of elastic fibers, as well as eosinophilic infiltration of the aortic sinus wall, thus suggesting an acquired etiopathogenesis.

Two-dimensional Doppler echocardiography can adequately predict sinus of Valsalva aneurysms. Cardiac catheterization confirms the diagnosis and the hemodynamic significance of the lesion, the associated cardiac anomalies, and the coronary anatomy can be precisely evaluated. A contrast-enhanced angio-CT scan or MRI, when feasible, can accurately assess aneurysm size, sinus of origin, aortic valve involvement, and the presence of associated cardiac abnormalities.

Rupture is the most frequent complication of a sinus of Valsalva aneurysm. An unruptured aneurysm of the sinus of Valsalva may cause right ventricular outflow tract obstruction, infective endocarditis, malignant arrhythmias, myocardial ischemia/infarction due to severe distortion of coronary ostia or compression of the coronary trunks, and dilatation of aortic annulus due to anatomical change. Our case presented a syndrome similar to a precardiac tamponade, due to a significant compression of her right atrium and due to the pericardial effusion. In such a case it was difficult to differentiate an unruptured aneurysm from a ruptured aneurysm or pseudoaneurysm of the sinus of Valsalva. Intrapericardial rupture or pseudoaneurysm carries a high rate of mortality, therefore, urgent surgical intervention is recommended in this subset of cases as in our case.

We decided to repair the affected noncoronary sinus, avoiding the possible drawbacks related to reduced aortic root pulsatile expansion [10]. The indication was prompted by the extremely large dimensions of the aneurysm [11]. Different techniques are employed to treat a sinus of Valsalva aneurysm, such as patch closure of the aneurysm orifice [3, 6, 8], valve-sparing operation [7], and Bentall operation [4, 5]. In two other cases in our experience with aneurysm of the right coronary sinus we employed a synthetic patch to close the aneurysm orifice associated with aortic valve replacement. In the reported case we preferred a patch closure of the orifice of the aneurysm since the aortic valve was normal and nonaortic annular dilatation was diagnosed. The aortic valve cusp should be evaluated for a good coaptation after repairing the coronary sinus. The closure of the orifice should be a remodeling procedure of the respective coronary sinus. In our case we preferred a double closure. The synthetic patch was implanted inside the aorta and then the aneurysm was opened, mainly to identify any communication with the cardiac chambers. Then the aneurysmatic sac was closed from outside above the orifice of the aneurysm.

In other cases in which the aortic root is dilated, the root replacement procedures should be applied according to established guidelines. In most of the cases the aortic annular ectasia is present and valve-sparing procedures might be applicable [7]; in other cases in which the aortic valve is diseased the Bentall procedure is indicated [4, 5].

## Conclusion

In conclusion we report the largest unruptured aneurysm of the noncoronary sinus of Valsalva, causing severe compression of the cardiac chambers, undergoing successful surgical correction.
